# A homozygous loss-of-function variant in *BICD2* is associated with lissencephaly and cerebellar hypoplasia

**DOI:** 10.1038/s10038-022-01060-x

**Published:** 2022-07-27

**Authors:** Ghada M. H. Abdel-Salam, Marian Girgis, Maha M. Eid, Inas S. M. Sayed, Mohamed S. Abdel-Hamid

**Affiliations:** 1 Clinical Genetics Department, Human Genetics and Genome Research Institute, National Research Centre, Cairo, Egypt; 2 Pediatric Department, Faculty of Medicine, Cairo University, Cairo, Egypt; 3 Human Cytogenetics Department, Human Genetics and Genome Research Institute, National Research Centre, Cairo, Egypt; 4 Orodental Genetics Department, Human Genetics and Genome Research Institute, National Research Centre, Cairo, Egypt; 5 Medical Molecular Department, Human Genetics and Genome Research Institute, National Research Centre, Cairo, Egypt

**Keywords:** Genetics research, Genetic testing

## Abstract

Developmental brain malformations are rare but are increasingly reported features of *BICD2-*related disorders. Here, we report a 2-year old boy with microcephaly, profound delay and partial seizures. His brain MRI showed lissencephaly, hypogenesis of corpus callosum, dysplastic hipocampus and cerebellar hypoplasia. Whole-exome sequencing identified a novel homozygous likely pathogenic variant in the *BICD2* gene, c.229 C > T p.(Gln77Ter). This is the first report of lissencephaly and cerebellar hypoplasia seen in a patient with homozygous loss-of-function variant in *BICD2* that recapitulated the animal model. Our report supports that *BICD2* should be considered in the differential diagnosis for patients with lissencephaly and cerebellar hypoplasia Additional clinical features of *BICD2* are likely to emerge with the identification of additional patients.

## Introduction

Dominant missense variants in the Bicaudal D2 Drosophila homolog 2 (*BICD2*) gene were initially described in autosomal dominant lower extremity-predominant spinal muscular atrophy 2 (SMALED2A;MIM#609797) [1] and its prenatal onset form (SMALED2B, MIM #618291) [2]. Subsequent reports linked heterozygous *BICD2* variants to hereditary spastic paraplegia [3] and developmental brain malformations [4, 5]. Recently, a homozygous *BICD2* variant was reported in a girl with Cohen-Like syndrome and abnormal gyral pattern [6].

Bicaudal D is required for the transport of mRNAs and other cellular cargoes as part of an essential pathway involving dynein and dynactin [7]. Loss-of-function in BicD2 was associated with defects in neuronal migration in the developing rat brain. It was postulated that defects in nuclear translocation that occur in the post-mitotic neuronal migration stage to be the mechanism of lissencephaly resulting from *BICD2* truncating variant [5].

We describe a novel lissencephaly and cerebellar hypoplasia disease and associate it with a recessive variant in the *BICD2* gene. Therefore, expanding the phenotypic spectrum of biallelic *BICD2*-associated disorders.

## Clinical report

**Fig. 1:**
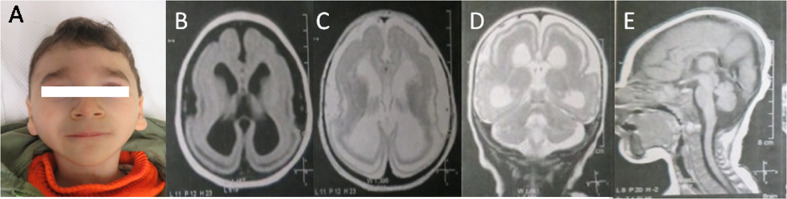
Our Patient at the age of 24 months (**A**) Note the thick eyebrows, upturned nostrils and microretrognathia. The brain MRI (**B**–**E**) showed lissencephaly with cell sparse zone, mild cerebellar hypoplasia, dysplastic hippocampus, hypoplastic corpus callosum and mild ventricular dilatation

Our patient is the second child of healthy consanguineous (first cousins) Egyptian parents. The pregnancy history was uneventful but prenatal ultrasound in the 30th week of gestation showed intrauterine growth retardation with small biparietal diameter. A male child was born at term by spontaneous vaginal delivery. His birth weight and OFC were 1800 g (−3 SD) and 30 cm (−3 SD), respectively. On physical examination at the age of 7 months, weight was 5800 g (−2.6 SD), length was 64 cm (−1.7 SD), and OFD was 35.5 cm (−5 SD). The EEG done at this time showed theta delta waves with minimal fast beta activity. He had plagiocephaly, almond shaped eyes, thick eyebrows, upturned nostrils and low set ears with thick ear lobules. Brain MRI (Fig. 1) showed lissencephaly, hypogenesis of corpus callosum, cerebellar hypoplasia. At the age of 24 months, his weight, length and OFC were 6500 g (−4.5 SD), 75 cm (−3.3 SD) and 36.5 cm (−8.4 SD), respectively. At that age, he developed partial seizures that showed good response to levetiracetam. He had profound psychomotor delay. Neurological examination showed spasticity of the extremities and increased deep tendon reflexes and positive Babiniski. Mild flexion contractures of the knee and clenched hands were noted. Prominent premaxilla, flat philtrum, thin lips, mandibular micrognathia and high arched palate were evident in oro-dental examination. Ultrasound examination of the abdomen revealed left moderate hydronephrosis. Bilateral optic atrophy was found in ophthalmologic examination. Complete blood picture showed normal results. He had male karyotype 46,XY. FISH (fluorescent in-situ hybridization) studies were performed, which ruled out 17p13.3 deletion.

**Fig. 2:**
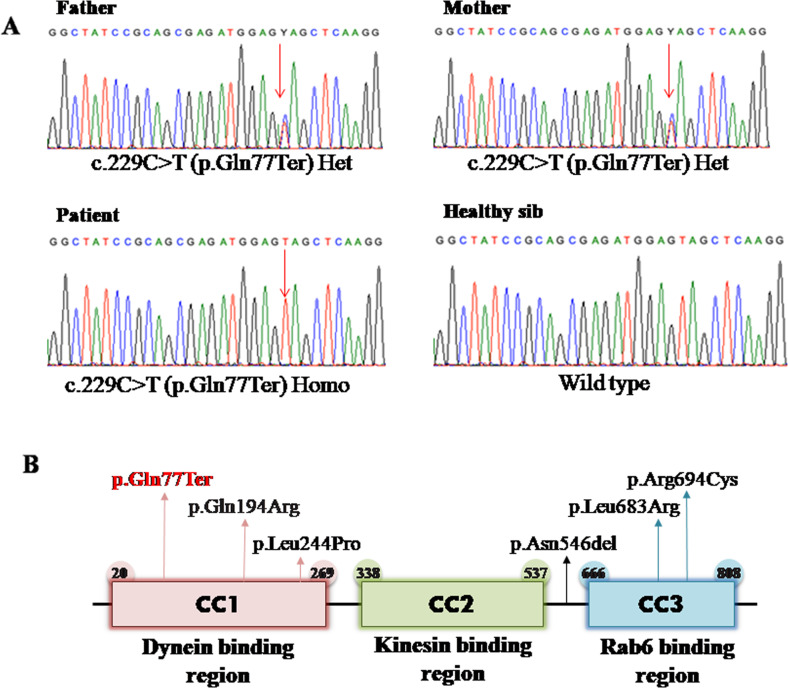
**A** Sequence chromatograms for c.229 C > T, p.(Gln77Ter) in the *BICD2* gene are shown for heterozygous parents, wild type sib and a homozygous patient. **B** Schematic diagram of BICD2 protein showing three coiled-coil domains with reported variants associated with *BICD2* continuum associated with brain anomalies

Exome sequencing (detailed method in Supplementary material) identified a homozygous stop gain variant in exon 1 of the *BICD2* gene NM_001003800.1: c.229 C > T: p.(Gln77Ter) as the likely causative gene of the patient’s phenotype. Based on the position of the variant, it is most likely led to nonsense-mediated decay. Unfortunately, we did not investigate the effect of the identified variant. The identified variant is not found in public genetic databases or our inhouse database of more than 500 exomes of cases with neurodevelopmental disorders and brain malformations. Segregation analysis using Sanger sequencing confirmed that both parents are heterozygous for the variant (Fig. 2). According to ACMG recommendations of variant classifications: the c.229 C > T p.(Gln77Ter) variant is detected as PVS1, PM2 and therefore classified as a “Likely Pathogenic”. No other disease-causing variants in previously reported genes, associated with his phenotypic spectrum, were identified. Moreover, the large-scale CNV (Supplementary material) data were further analyzed and no disease-causing large duplications or deletions within coding regions were identified.

## Discussion

The identified variant p.(Gln77Ter) is new and absent from the Genome Aggregation Database. It was evidenced that pathogenic variants in *BICD2* are extremely rare in the population, predicted to be damaging by most tools, and occur in specific hotspots within key *BICD2* functional domains [8]. Furthermore, WES did not identify any variant(s) in any of the OMIM genes with an acknowledged disease association (including *VPS13B* gene). Although *BICD2* is essential for the proper development of the cerebral cortex [5] but there have been no other clinical reports of individuals with loss of-function variants in *BICD2* showing lissencephaly and cerebellar hypoplasia. However, lissencephaly and cerebellar hypoplasia are consistent with that observed after BICD2 knockdown in mice showing defects in laminar organization of the cerebral cortex, hippocampus and cerebellar cortex, indicative of radial neuronal migration defects. Cell-specific inactivation of BICD2 in astrocytes and neuronal precursors revealed that radial cerebellar granule cell migration is non-cell-autonomous and intrinsic to cerebellar Bergmann glia cells [9, 10]. Therefore, we considered *BICD2* to be a convincing candidate gene in the context of lissencephaly and cerebellar hypoplasia. The absence of homozygous loss of function *BICD2* variants in the healthy family members supports the clinical relevance of *BICD2*.

Recently, biallelic variant c.731 T > C p.(Leu244Pro) in *BICD2* was described in a girl with abnormal gyral pattern in fronto-temporo-parietal regions [6] (Table 1). The girl displayed additionally moderate intellectual disability and Cohen-like features [6]. In comparison, our patient showed congenital microcephaly, profound delay, seizures, lissencephaly and cerebellar hypoplasia. Unlike the patient with Cohen-like features, our patient showed spasticity and developed contracture deformities and did not show neutropenia. Interestingly, a heterozygous missense variant c.2080 C > T, p.(Arg694Cys) was reported in two unrelated patients with mild perisylvian polymicrogyria, and mild cerebellar vermis hypoplasia [4]. Moreover, a *BICD2* nonsense variation p.(Lys775Ter) was identified in a boy with lissencephaly and subcortical band heterotopia [5]. These heterozygous variants are located within the highly conserved CC3 domain of BICD2 (Table 1). Nevertheless, the heterozygous missense variants within the CC1 domain were not associated with abnormalities of cortical development but even showed a milder course of SMALED2A and a higher frequency of foot deformities [8]. Indeed, a larger cohort is required to draw conclusions regarding genotype-phenotype correlations.

**Table 1:** The clinical findings and variants identified in patients with *BICD2* and brain anomalies

	Fiorillo et al. [13]	Ravenscroft et al. [4]		Koboldt et al. [8]		Storbeck et al. [2]	Tsai et al. [5]	Caglayan et al. [6]	This study
		Patient 1	Patient 2	Patient 1	Patient 2				
Gender	Male	Male	Male	Female	Male	Female	Male	Female	Male
Age at last examination	7 Y	4 Y	45 days	12 Y	6 Y	4 M	4 Y	12 ^7^/_12_ Y	2 Y
Microcephaly	−	+	−	−	−	−	+	+	+
Abnormality of the ear	−	−	−	+	−	−	−	−	+
Almond shaped eyes	−	−	−	−	−	−	−	+	+
Micrognathia	−	+	+	−	+	−	−	+	+
High arched palate	−	−	−	−	−	−	−	+	+
DQ/IQ	Normal	Severe	NA	Severe	Severe		Severe	Moderate	Profound
Arthrogryposis/contracture deformities	+	+	+	+	+	+	−	−	+ (contractures in the knees)
Seizures	−	−	−	+	+	−	−	−	+
Lissencephaly/									
Pachygyria	−	−	−	−	−	−	+	+	+
Hippocampus	−	−	−	NA	NA		−	−	+
Hypogenesis of corpus callosum	−	+	+	+	+	+	−	−	+
Polymicrogyria	−	+	+	−	−	−	−	−	−
Cerebellar hypoplasia	+	+	+	−	−	−	−	−	+
White matter loss	−	+	+	+	+(near complete absence)	−			
Ventriculomegaly	−	+	+	−	+ (marked)	−	+	−	+
Peripheral neuropathy	+	+	+	NA	NA	+	−	−	−
BICD2 domain	CC3	CC3	CC3	Outside CC2	Outside CC2	CC1	CC3	CC1	CC1
Zygosity	Heterozygous	Heterozygous	Heterozygous	Heterozygous	Heterozygous	Heterozygous	Heterozygous	Homozygous	Homozygous
Variant	c.2048 T > G (p.Leu683Arg)	c.2080 C > T (p.Arg694Cys)	c.2080 C > T (p.Arg694Cys)	c.1636_1638delAAT (p.Asn546del)	c.1636_1638delAAT (p.Asn546del)	c.581 A > G (p.Gln194Arg)	c.2323 A > T (p.Lys775Te)	c.731 T > C (p.Leu244Pro)	c.229 C > T (p.Gln77Ter)

*NA* not available, *M* month, *Y* year, *CC1* coiled coil domain 1, *CC2* coiled coil domain 2, *CC3* coiled coil domain 3

Lissencephaly and cerebellar hypoplasia noticed in our patient appeared similar to those with *LIS1* variants. This is not surprising as *LIS1* interacts with the dynein/dynactin complex and BICD2 to recruit cellular structures [11]. In the mean time, these brain MRI features may overlap with *RELN*-mutated patients phenotype. However, the cortical migration defect was more severe in our patient than in *RELN*-mutated patients. In addition, our patient had mild cerebellar hypoplasia unlike *RELN*-mutated patients who had profoundly hypoplastic and dysplasic cerebellum with no identifiable folia [12].

Our study provides valuable findings into human developmental brain malformations disorders associated with definitive loss-of function variants in *BICD2*.

## Supplementary information

Whole exome sequencing quality metrics

## Data Availability

The data that support the findings of this study are available with the corresponding authors upon reasonable request.
