# Supplementary material for: A homozygous loss-of-function variant in BICD2 is associated with lissencephaly and cerebellar hypoplasia
Source: J Hum Genet. 2022 Jul 27;67(11):669–73. doi: 10.1038/s10038-022-01060-x (PMC9592554; doi:10.1038/s10038-022-01060-x)
Supplement: Supplementary file 1 — Whole exome sequencing quality metrics [file 10038_2022_1060_MOESM1_ESM.doc]

**Whole exome sequencing quality metrics**

Genomic DNA is enzymatically fragmented, and target regions are enriched using DNA capture probes. These regions include approximately 41 Mb of the human coding exome (targeting > 98% of the coding RefSeq from the human genome build GRCh37/hg19), as well as the mitochondrial genome. The generated library was sequenced on an Illumina platform to obtain at least 20x coverage depth for > 98% of the targeted bases. Obtained sequences were aligned to UCSC human genome GRCh37/hg19 and variants were verified through the GATK pipeline. Annotation of variants is done using BaseSpace Variant Interpreter Server. Identified variants are checked against public genetic databases like Genome Aggregation Database (gnomAD, <https://gnomad.broadinstitute.org/>), 1000 Genomes ([www.1000genomes.org](http://www.1000genomes.org/)), and dbSNP (<http://www.ncbi.nlm.nih.gov/SNP/>) and also our inhouse database. Variants with minor allele frequency of less than 1% in gnomAD database, and disease-causing variants reported in HGMD®, in ClinVar are evaluated. The investigation for relevant variants is focused on coding exons and flanking +/-10 intronic nucleotides of genes with clear gene-phenotype evidence. All potential patterns for mode of inheritance are considered. In addition, provided family history and clinical information are used to evaluate identified variants with respect to their pathogenicity and disease causality.

Copy number variants were analyzed by ExomeDepth software version 1.0.7. The initial BAM files were realigned and the base quality scores were recalibrated. After marking the duplicates the final set of alignment data required for computational CNV prediction were generated. The genome builds referencesequence used was hg19.
